# Developing a suicide prevention action plan in Kisumu County, Kenya

**DOI:** 10.1002/puh2.112

**Published:** 2023-07-31

**Authors:** Sarah Atieno Ouma, Florina Frey, Gregory Ganda, Beryl Annie Arogo, Douglas Onyango Otieno, Paul Andrä, Rick Peter Fritz Wolthusen

**Affiliations:** ^1^ School of Medicine Maseno University Kisumu Kenya; ^2^ School of Medicine University Hospital Düsseldorf Düsseldorf Germany; ^3^ School of Medicine University Hospital Dresden Dresden Germany; ^4^ Kisumu County Department of Health and Sanitation County Government of Kisumu Kisumu Kenya; ^5^ Mental Health Mashinani Nairobi Kenya; ^6^ Tinada Youth Organization Kisumu Kenya; ^7^ On The Move e.V. Dresden Germany; ^8^ Harvard Kennedy School Cambridge USA; ^9^ Department of Psychiatry and Behavioral Sciences Duke University Medical Center Durham North Carolina USA

**Keywords:** mental health, middle‐income country, policymaking, suicide prevention

## Abstract

Suicide prevention is a complex and context‐dependent challenge. About 75% of deaths by suicide occur in low‐ and middle‐income countries; yet, most current suicide prevention strategies build upon data from high‐income countries (HIC). The Kisumu County Government (KCG) in Kenya recognized the need for a suicide prevention action plan. In the absence of a Kenyan national suicide strategy, it also did not solely depend on recommendations from HIC. The KCG therefore convened a multidisciplinary workgroup with stakeholders from various sectors that led the development of the Kisumu County Suicide Prevention Action Plan (KCSPAP). The team utilized a mixed‐method approach (literature review, a desk review of mental health indicators and death certificates, focus group discussions, and key informant interviews) identifying the following: (a) magnitude and variations of suicide cases (higher number of suicide completions compared to attempts; the leading method was organophosphate poisoning though the suicide method often was not specified); (b) protective and risk factors (male gender, being between 19 and 45 years of age, and being married); (c) community perceptions of suicide (taboo topic; associated with negative spirits; community members were divided on suicide decriminalization); (d) potential solutions (need for data collection, awareness creation; scale‐up of traditional and nontraditional mental health approaches). Given the importance of a public health perspective on suicide prevention, the data in the KCSPAP are organized in a public health prevention framework that builds on a data collection framework. The KCSPAP was handed over to the KCG in mid‐2020; different recommendations have been implemented since. The KCSPAP is an example of policymaking based on local knowledge. This homegrown policymaking approach has multiple benefits and can be used by stakeholders locally and in other countries.

## INTRODUCTION

About 800,000 people die by suicide every year globally [1]. Six African countries (the Central African Republic, Eswatini, Lesotho, Mozambique, South Africa, and Zimbabwe) are among the 10 countries with the highest age‐standardized suicide rates per 100,000 people worldwide in 2019 [2]. Kenya has an age‐standardized suicide rate of 11 per 100,000 people [3]. This number is likely an underestimate due to stigma and criminalization of suicides [4].

**FIGURE 1:**
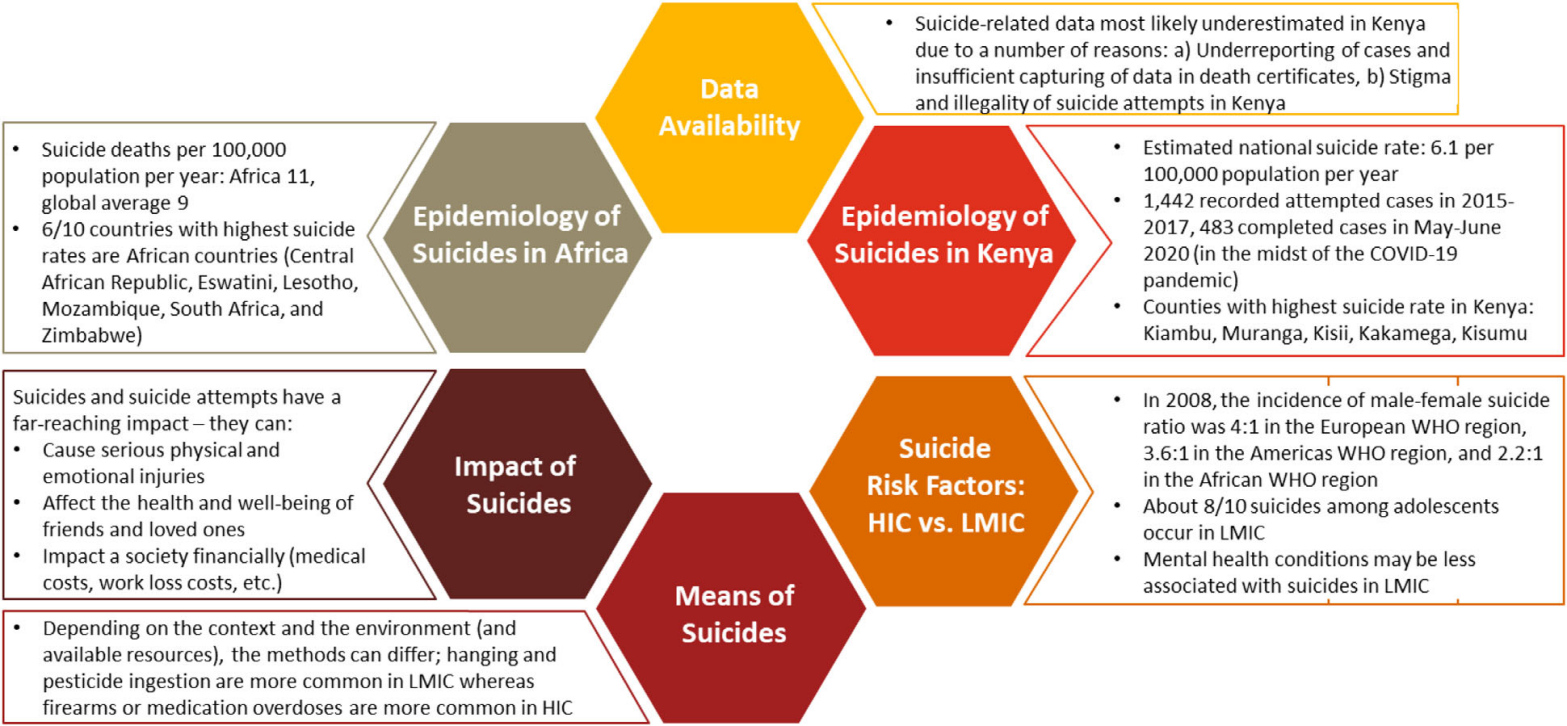
Description of the epidemiology of suicides, data availability, means of suicides, the impact of suicides, and risk factors for suicides in high‐income countries (HIC) and low–middle‐income countries (LMIC).

Three out of four suicides happen in low‐ and middle‐income countries (LMIC). Conversely, most of the research regarding suicides is conducted in high‐income countries (HIC). Most of these findings from HIC are not generalizable to LMIC (see Figure 1 for a detailed description of the epidemiology of suicides, data availability, means of suicides, the impact of suicides, and risk factors for suicides) [5]. For example, a systematic review and meta‐analysis of 37 case–control studies from 23 countries demonstrated that having a mental disorder and a history of self‐harm are the strongest associated risk factors for suicide. The presence of mental illnesses may contribute less to suicide attempts in LMIC than in HIC [6]. Further, the means of suicide differ between regions and countries: hanging and pesticide ingestion are more common in LMIC, whereas firearms or medication overdoses are more common in HIC. According to the World Health Organization, only 38 countries had a national suicide prevention strategy in 2018; and Namibia was the only country in Africa [7].

The COVID‐19 pandemic impacted the mental health of Kenyan citizens significantly. There were 483 cases of suicides, which were recorded in just 3 months of 2021, up from 421 in all of 2017 [8]. Kisumu County, one of the 47 counties, had the fourth‐highest absolute number of suicide cases in Kenya during the COVID‐19 pandemic. In 2022, it was estimated that the suicide‐specific mortality rate in Kisumu County was 14.7 per 100,000 populations per year [9]. However, even before the pandemic, the Kisumu County Government (KCG) recognized the urgency of a suicide prevention strategy. This commentary discusses how the Kisumu County Suicide Prevention Action Plan (KCSPAP) was developed, highlights some key findings, and reflects on the first achievements reached, and the potential benefits of policymaking based on local knowledge.

## THE KISUMU COUNTY SUICIDE PREVENTION ACTION PLAN

In Kenya, the national government is responsible for policy and framework formulation, and the county governments are responsible for adopting and implementing these policies (refer to Table 1 for more information about Kenya's social, economic, and health indicators). However, the National Kenyan Suicide Prevention Strategy was only published in 2022. Due to a lack of a national suicide prevention strategy in 2020, the KCG could not just customize an existing national strategy. In order to become proactive and fill this vacuum, the KCG convened a multidisciplinary workgroup with stakeholders from various sectors that led the development of a suicide prevention action plan based on Kisumu County‐specific data. The workgroup focused on four thematic core areas around suicides (magnitude and variations of suicide cases, risks and protective factors, community perceptions, and potential solutions to decrease the number of suicides). The team utilized a mixed‐method approach (literature review, desk review of mental health indicators and death certificates, eight focus group discussions, and nine key informant interviews). All data were collected in January 2020 and analyzed between February and March 2020.

**TABLE 1:** Economic and health and mental health indicators for Kenya and socioeconomic factors affecting suicide in Kenya.

Economic indicators	Health indicators	Mental health indicators
Annual average growth: 5.9% (2010–2018), slowed down with COVID‐19 GDP: 110 billion USD (2021) Lower–middle income country Fast growing economy before COVID‐19 5/13 Kenyans live in poverty (38.6%, 2021)	Average life expectancy: 66.1 years (2018) Maternal mortality rate: 362/100,000 live births; neonatal mortality rate: 22/1000 live births Disability adjusted life years: 17,856,955 (2017) Leading causes of death: HIV/STIs, cardiovascular disease, respiratory infections/TB, cancer	DALY for mental disorders: 656,588 1.9 million cases of depression, ranking 4th in Africa 1 in 4 Kenyans suffers from a mental illness in their lifetime Outpatient care: 20%–25% of patients present symptoms of a mental health condition

Socioeconomic factors affecting suicide in Kenya		
Low growth of domestic GDP, high levels of unemployment leading to poverty, high population growth rate, rural–urban migration, insufficient planned urbanization, low literacy levels, deforestation and inequitable land distribution, lack of mental healthcare access, and stigma affecting perceptions of suicide		

Abbreviations: GDP, gross domestic product; HIV, human immunodeficiency virus; STI, sexually transmitted illness; TB, tuberculosis; DALY, disability adjusted life years.

In terms of magnitude and variation of suicides, the analysis revealed that the number of completed suicide cases in Kisumu County was higher than the number of suicide attempts officially reported. A significant number of death certificates did not specify the method of suicide. However, for cases of suicide with specified methods, organophosphate poisoning was the leading cause. Regarding risk and protective factors, community members with the following characteristics were at higher risk: male gender, being between 19 and 45 years of age, and being married. Notably, there was no gender difference regarding the risk for suicide attempts. Additionally, the focus group participants and interviewees identified the following risk factors for suicide: marital and relationship issues, family feuds, economic and job insecurity, poverty, substance use, land disputes, history of sexual trauma, and “Satan” (i.e., “The Devil”). In terms of perception of suicides, suicides were often a taboo topic and associated with negative spirits. Community members described the need to perform rituals after suicide attempts and completions to remove bad spirits and prevent additional suicides. Community members were divided over whether suicide attempts should remain illegal. Lastly, regarding potential solutions, the need for data collection, awareness creation, a scale‐up of traditional and nontraditional (i.e., services offered by trained community members) mental health services, and the implementation of restrictions around organophosphates were identified.

## STRATEGIC PREVENTION FRAMEWORK

**FIGURE 2:**
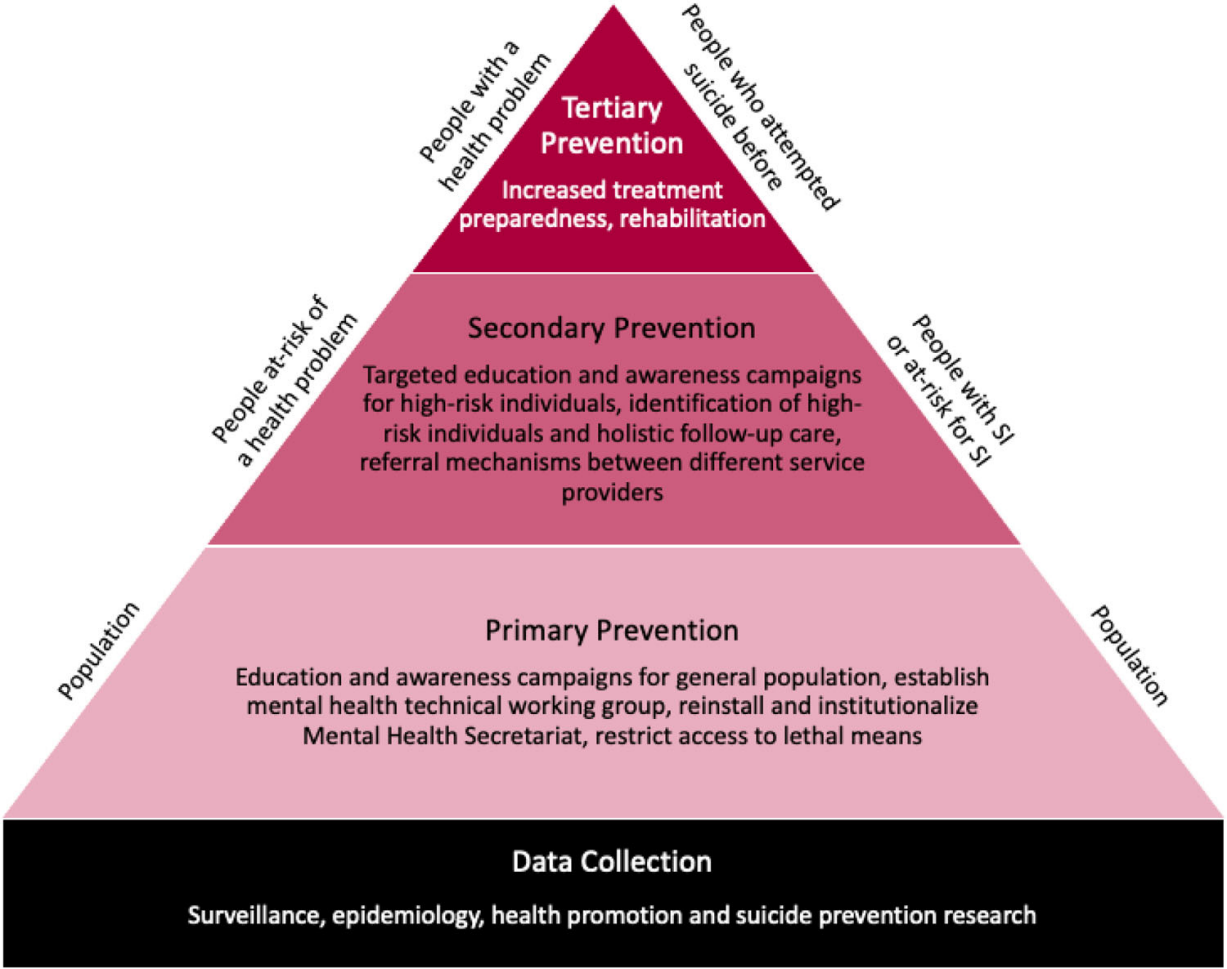
The Kisumu County Suicide Prevention Action Plan (KCSPAP) is built on two frameworks: a data collection framework and a prevention framework.

The findings of the study were conceptualized by using the data collection framework and the prevention framework (see Figure 2). The data collection framework includes recommendations for data collection for surveillance, epidemiology as well as for health promotion and disease prevention research purposes. Accurate data collection will allow the executive (KCG) and legislative (Kisumu County Assembly) to allocate resources for suicide prevention and mental health overall. Primary prevention, which aims to prevent disease or injury before they occur on a population level, includes activities such as education, awareness campaigns about mental health and suicide, restricting access to legal means for suicide, and recommendations for system strengthening and governance (i.e., the institutionalization of a mental health technical working group within the government). Secondary prevention, which targets people at risk for a health problem before the onset of symptoms, includes more comprehensive education and awareness campaigns for certain professions (such as teachers, law enforcement, and journalists) and expansion of screening for suicide by conventional mental health providers and nonconventional trained stakeholders (such as religious leaders). Tertiary prevention targets community members who previously attempted to die by suicide and include county‐specific recommendations regarding expanding (mental) health services and biopsychosocial rehabilitation opportunities (see Table 2 for more details).

**TABLE 2:** Strategies discussed in the Kisumu County Suicide Prevention Action Plan (KCSPAP) sorted by prevention levels.

Levels of prevention	Strategies and activities	
Primary prevention	Strategy 1	Education and awareness campaigns to sensitize the population
	Strategy 2.1	Improve governance by setting up a technical mental health working group
	Strategy 2.2	Reestablish and institutionalize the Mental Health Secretariat
	Strategy 3	Restrict access to legal means for suicide
Secondary prevention	Strategy 4.1	Education and awareness campaigns to sensitize professionals working with target populations at‐risk, e.g., teachers and law enforcement
	Strategy 4.2	Education and awareness campaigns to reduce the number of copycat suicides
	Strategy 4.3	Education and awareness campaigns to target professional groups at risk for suicide
	Strategy 5	Develop the mental health workforce that can identify and follow‐up on cases of mental illness and suicidal ideation in the community
	Strategy 6	Strengthen the collaboration between conventional medical practitioners and alternative medical professionals
	Strategy 7	Customize training modules to allow holistic mental health counseling and suicide prevention
Tertiary prevention	Strategy 8	Improve readiness to respond to suicide cases and cases of organophosphate poisoning
	Strategy 9	Offer medical and biopsychosocial rehabilitation

*Note*: The strategies were designed based on the findings of the county‐specific qualitative and quantitative data collection.

## LESSONS FROM THE SUICIDE PREVENTION ACTION PLAN

The KCSPAP was handed over to the KCG in mid‐2020, which has been working on implementing different recommendations. To date, the qualitative evidence collected through the KCSPAP provided the foundation for effective advocacy work, which resulted in financial allocations toward mental health through the Kisumu County Assembly. Additionally, a multisectoral mental health stakeholder forum was established for sustainability reasons, and a mental health technical working group within the government was revived.

At a time of rising numbers of suicide cases, the KCG could not borrow from a national suicide prevention strategy. However, the KCSPAP, being the first of its kind in Kenya, was able to fill the strategy vacuum in the county. As the KCSPAP is more context‐sensitive, these county data on protective and risk factors could be tapped to inform future local and national action plans. The work on the KCSPAP created momentum for the belief that complex challenges, such as suicide prevention, can be targeted effectively locally when all relevant stakeholders own the process and work on a shared mission. Through sharing of information, the work on the KCSPAP also allowed for building trust among stakeholders, such as government officials, mental health professionals, and nonconventional practitioners, including religious leaders. The discussions around suicide prevention also raised awareness about mental health conditions and the need to integrate mental health programs into existing activities targeting psychosocial health determinants (i.e., programs focusing on youth unemployment or gender‐based violence).

Following the example of the KCSPAP, stakeholders from other counties in Kenya, other countries on the African continent, or from other LMIC may engage in bottom‐up strategy development based on professional, scientific, and, equally important, local knowledge rather than in implementing top‐down policies only. Local knowledge reflects the community members’ worldviews and lived experiences referring to knowledge accumulated over many generations. Research studies from other LMIC, such as Indonesia [10], shore up the benefits of including local knowledge, through community participation and co‐creation, in policy design and implementation processes. Some benefits are a decrease in the gap among those designing, implementing, and benefitting from policies, an increase in the fitness of policies, and opportunities for community members to provide more immediate feedback.

## CONCLUSION

The KCG recognized the increasing number of suicide cases as a public health challenge in early 2020. The KCG tasked a workgroup with the development of a KCSPAP that was informed by research around four thematic core areas (magnitude and variations of suicide cases, risks and protective factors of suicide, community perceptions of suicide, and potential solutions to decrease the number of suicides). The qualitative and quantitative data collected were conceptualized with the help of two public health frameworks: the data collection framework and the prevention framework. The KCSPAP filled a strategy vacuum and identified county‐specific suicide protective and risk factors. The KCSPAP is an example of policymaking based on local knowledge. This local, homegrown policymaking approach has multiple benefits and can be used by stakeholders in a similar situation to the KCG.

## AUTHOR CONTRIBUTIONS

*Data curation; project administration; validation; writing—original draft; writing—review and editing*: Sarah Ouma Atieno. *Writing—original draft; writing—review and editing*: Florina Frey and Paul Andrä. *Writing—review and editing*: Gregory Ganda. *Data curation; project administration; writing—review and editing*: Beryl Annie Arogo. *Project administration; writing—review and editing*: Douglas Onyango Otieno. *Conceptualization; data curation; formal analysis; investigation; methodology; project administration; supervision; writing—original draft; writing—review and editing*: Rick Peter Fritz Wolthusen.

## CONFLICT OF INTEREST STATEMENT

No conflict of interest.

## FUNDING INFORMATION

There was no funding in the development for this paper.

## ETHICS STATEMENT

The research was exempted through the Harvard Kennedy School IRB.

## Data Availability

The data that support the findings of this study are available from the corresponding author upon reasonable request.

## References

[1] Bachmann S . Epidemiology of suicide and the psychiatric perspective. Int J Environ Res Public Health. 2018;15(7):1425. 29986446 10.3390/ijerph15071425 PMC6068947

[2] World Health Organization, African Region . Reversing Suicide, Mental Health Crisis in Africa [Internet]. World Health Organization, African Region; 2022 [cited 18 Oct 2022]. Available from: https://www.afro.who.int/news/reversing‐suicide‐mental‐health‐crisis‐africa

[3] World Health Organization . Suicide rate estimates, age‐standardized estimates by country. In: Global Health Observatory Data Repository 2021 [Internet]. World Health Organization; 2021 [cited 2 Jan 2023]. Available from: https://apps.who.int/gho/data/node.main.MHSUICIDEASDR?lang=en

[4] National Council for Law Reporting with the Authority of the Attorney‐General . Laws of Kenya, Penal Code, Chapter 63.. In: International Labour Organization 2012: Laws of Kenya [Internet]. National Council for Law Reporting with the Authority of the Attorney‐General; 2012 [cited 18 Oct 2022]. Available from: https://www.ilo.org/dyn/natlex/docs/ELECTRONIC/28595/115477/F‐857725769/KEN28595.pdf

[5] Knipe D , Williams AJ , Hannam‐Swain S , et al. Psychiatric morbidity and suicidal behaviour in low‐ and middle‐income countries: a systematic review and meta‐analysis. PLoS Med. 2019;16(10):e1002905. 31597983 10.1371/journal.pmed.1002905 PMC6785653

[6] Favril L , Yu R , Uyar A , et al. Risk factors for suicide in adults: systematic review and meta‐analysis of psychological autopsy studies. Evid Based Ment Health. 2022;25:148‐155. 36162975 10.1136/ebmental-2022-300549 PMC9685708

[7] World Health Organization . National Suicide Prevention Strategies: Progress, Examples and Indicators [Internet]. World Health Organization; 2018 [cited 2 Jan 2023]. Available from: https://apps.who.int/iris/bitstream/handle/10665/279765/9789241515016‐eng.pdf

[8] Wambui V . Out of 421 suicide cases in Kenya, 330 involved men. People Daily Kenya [Internet]. 2019 [cited 18 Oct 2022]. Available from https://www.pd.co.ke/news/why‐the‐suicide‐ogre‐stalks‐many‐4485/

[9] Ongeri L , Larsen DA , Jenkins R , et al. Community suicide rates and related factors within a surveillance platform in Western Kenya. BMC Psychiatry. 2022;22:7. 34983463 10.1186/s12888-021-03649-6 PMC8729019

[10] Nugroho K , Carden F , Antlov H . Local Knowledge Matters: Power, Context and Policy Making in Indonesia. 1st ed. Bristol University Press; 2018.

